# Timely approaches to identify probiotic species of the genus *Lactobacillus*

**DOI:** 10.1186/1757-4749-5-27

**Published:** 2013-09-24

**Authors:** Stefan R Herbel, Wilfried Vahjen, Lothar H Wieler, Sebastian Guenther

**Affiliations:** 1 Centre for Infection Medicine, Institute of Microbiology and Epizootics, Freie Universität Berlin, Robert-von-Ostertag-Str. 7-13, Berlin, 14163, Germany; 2 Department of Biology, Chemistry, Pharmacy, Freie Universität Berlin, Takustr. 3, Berlin, 14195, Germany; 3 Institute of Animal Nutrition, Freie Universität Berlin, Königin-Luise-Str. 49, Berlin, 14195, Germany

## Abstract

Over the past decades the use of probiotics in food has increased largely due to the manufacturer’s interest in placing “healthy” food on the market based on the consumer’s ambitions to live healthy. Due to this trend, health benefits of products containing probiotic strains such as lactobacilli are promoted and probiotic strains have been established in many different products with their numbers increasing steadily. Probiotics are used as starter cultures in dairy products such as cheese or yoghurts and in addition they are also utilized in non-dairy products such as fermented vegetables, fermented meat and pharmaceuticals, thereby, covering a large variety of products.

To assure quality management, several pheno-, physico- and genotyping methods have been established to unambiguously identify probiotic lactobacilli. These methods are often specific enough to identify the probiotic strains at genus and species levels. However, the probiotic ability is often strain dependent and it is impossible to distinguish strains by basic microbiological methods.

Therefore, this review aims to critically summarize and evaluate conventional identification methods for the genus *Lactobacillus*, complemented by techniques that are currently being developed.

## Introduction

Members of the genus *Lactobacillus* are Gram positive, acid tolerant, facultative anaerobic and fermentative bacteria with low G + C content belonging to the phylum Firmicutes [1]. They are common in food related habitats such as wine, milk, meat, fruits, vegetables and cereal grains and are often used as starter cultures for food fermentation processes [2]. Additionally, some members of the genus *Lactobacillus* are naturally associated with mucosal surfaces, residing in parts of the intestinal tract, vaginal and oral cavity of humans and animals [3]. Lactobacilli have been used for millennia for the preservation of food, e.g. cured meat such as salami or pickeled vegetables such as sauerkraut and olives. They are also very common as starter and adjunct cultures of dairy products such as yoghurt and cheese [4]. Food which is claimed to have a beneficial effect on the consumer’s health by using microbial dietary ingredients are known as functional, designer or fortified food containing probiotics [5].

Over the last decades a wide range of these functional products containing probiotics have been made available in the market which is fostered by the current trend of consuming healthy food in order to prevent illness [6]. Besides bifidobacteria, lactobacilli are currently among the most important probiotics and species like *Lactobacillus casei* are widely used in food supplements and lactic beverages such as Yakult® (Yakult, Germany) or Actimel® (Danone, Germany) [7].

The word “probiotic” is a composite of the Latin preposition pro (“for”) and the Greek adjective of the noun βίoς (bios, “life”)” [8]. Therefore, the viability of probiotic bacteria within products is crucial for the beneficial effects they intend to offer to the consumer’s health. For example in order to offer beneficial effects to the host, 10^6^ to 10^8^ colony-forming units (cfu) per ml are needed as viable bacteria till the end of storage time [9,10]. Nevertheless, several studies showed low survival rates of utilized probiotic strains within storage time of products [9].

For quality management reasons and in order to adhere to the European Health Claims Regulations (EC, No 1924/2006 of the European Parliament and of the Council of 20^th^ December 2006) fast and reliable tools are needed to identify and quantify probiotic strains used in a product [7,11].

Despite the large economic impact of lactobacilli most of the assays currently used for their identification are classical microbiological methods which are time consuming and not easy to standardize. Furthermore, phenotypic identification may fail due to misidentification [12]. These basic phenotypic methods include morphology, Gram staining and biochemical tests such as fermentation of carbohydrates or growth at varying temperatures and salt concentrations [12]. Morphology screening for differentiation seems problematic, as it is known that lactobacilli have diverse morphotypes within the same species [13]. Fortunately, for species-specific identification of strains based on physiological properties other modern tools have become available over the last years such as the API system from Biomérieux (France), the Diatabs system (Rosco, Denmark) or the BIOLOG GP MicroPlate System (BIOLOG Inc., USA). Additionally, rapid identification tools based on genomic features of lactobacilli include 16S or 16S-23S rDNA (ITS)-PCR and quantitative real time PCR analysis or proteomic analysis using MALDI-TOF MS [7] (Table 1).

**Table 1:** Experiment duration and detection level for each method

**Method**	**Duration (h**^***a***^**)**	**Time in total (h)**		**Detection at the level of…**			**Quantification**
		**Culture-dependent**	**Culture-independent**^***b***^	**Genus**	**Species**	**Subspecies**	
Morphology	*^c^	~ 48	^/ *d*^	-^*e*^	-	-	-
FTIR	* + 1 h (analyzation)	~ 49	^/^	+^*f*^	+	-	-
MALDI-TOF MS	* + 1 h (analyzation)	~ 49	^/^	+	+	+	-
API 50 CHL	* + 48 h (incubation)	~ 96	^/^	+	+	-	-
BIOLOG	* + 25 h (24 h incubation of AN MicroPlate^TM^ + 1 h analyzation)	~ 72	^/^	+	+	-	-
16S/23S rRNA PCR + sequencing	* + 31 h (4 h DNA isolation + 3 h PCR + 24 h sequencing)	~ 79	~ 31	+	+	-	-
PCR-DGGE	* + 7 h (4 h DNA isolation + 3 h PCR + electrophoresis)	~ 55	~ 7	+	+	-	(+)^*g*^
RAPD	* + 7 h (4 h DNA isolation + 3 h PCR + electrophoresis)	~ 55	~ 7	+	+	+	-
SSCP	* + 7 h (4 h DNA isolation + 3 h PCR + electrophoresis)	~ 55	~ 7	+	+	-	-
MLST	* + 31 h (4 h DNA isolation + 3 h qPCR + 24 h sequencing)	~ 79	~ 31	+	+	+	-
qPCR	* + 7 h (4 h DNA isolation + 3 h qPCR)	~ 55	~ 7	+	+	-	+
SSR	* + 7 h (4 h DNA isolation + 3 h PCR + electrophoresis)	~ 55	~ 7	+	+	+	-
WGS	* + 4 h DNA isolation + 36 h Sequencing, annotation, etc.)	~ 88	~ 40	+	+	+	-

^*a*^ h, hour(s).

^*b*^ duration exclusive cultivation of the strains (48 h).

^*c*^ *, isolation of the strains by plating on different media (MRS broth [Roth, Germany [14]], LBS agar [Becton Dickinson, USA [15]], COL and CHOC plates [Sarstedt, Germany]).

^*d*^ /, identification of the strains not possible by working culture-independent.

^*e*^ -, detection at the level of… is not possible.

^*f*^ +, detection at the level of… is possible.

^*g*^ (+), a limited quantification is possible using PCR-DGGE.

This review aims to evaluate classical microbiological identification methods of the genus *Lactobacillus* complemented by newly developed molecular techniques. As probiotic lactobacilli are successfully used currently, the first chapter of the review will deal with a short overview on probiotic health benefits. In the second part different phenotypic, physicochemical and genotypic methods will be discussed. The detection level in terms of genus, species, subspecies and strain specificity will also be in focus. As probiotic effects are often strain dependent the latter is of utmost importance.

## Probiotics

### Definition

In 1989 Fuller defined probiotics for animals as ‘a live microbial feed supplement which beneficially affects the host animal by improving its intestinal microbial balance’ [16]. By using this definition he assumed probiotics as live bacteria which have a beneficial effect on the host. Later on Schrezenmeir and de Vrese (2001) amended the definition as ‘a preparation of or a product containing viable, defined microorganisms in sufficient numbers, which alter the microbiota (by implantation or colonization) in a compartment of the host and by that exert beneficial health effects in this host’ [17]. In 2010 the World Health Organization (WHO) defined probiotic strains as ‘live microorganisms that, when administered in adequate amounts, confer a health benefit on the host’ [18]. In general, bacteria have to comply with the following selection criteria to be cited as ‘probiotics’ [19,20]:

be of human or bovine origin and non-pathogenic,

sustain integration into food in high cell counts,

maintain their viability throughout shelf-life of the product,

be resistant towards bile and acid juice and withstand transition through the GI tract,

be an antagonist towards pathogenic bacteria in the gut,

offer health benefits.

Probiotic bacteria offer a wide range of beneficial effects. They are able to decrease the duration of diarrhea, reduce allergic syndromes, deliver various bacteriocins and lower the pH subsequently inhibiting invasion of pathogens such as *Salmonella* spp. or *Escherichia coli*[21].

As mentioned above, probiotics are widely used in fermented food and feed due to their presumed beneficial effects on host’s health. However, these health benefits are often strain specific, therefore unambiguous identification to species and strain level is important [22]. This has been found by using strains singly and in combination with other strains resulting in a reduced or suppressed effect of their benefits when used in combination [23]. Thus, each new combination of probiotic strains has to be studied to avoid the use of non-functional probiotic bacteria [23].

Most probiotic organisms currently used in food for humans belong to either the genus *Lactobacillus* or *Bifidobacterium*. Bifidobacteria are Gram positive, non-motile, non-sporulating, anaerobic and hetero-fermentative bacteria with a high G + C content. Members of the genus *Lactobacillus* are also Gram positive, non-motile and non-sporulating; however, they are acid tolerant and facultative anaerobes, homo- or heterofermentative and have a low G + C content.

Other bacteria used as probiotics in human and animal nutrition include *Escherichia coli* strain Nissle 1917, *Lactococcus lactis*, *Streptococcus thermophilus* and *Enterococcus faecium* (Wysong, USA). Additionally, for more than three decades *Bacillus toyonensis* sp. nov. (formerly described as *Bacillus cereus* var *toyoi*, [24]) has been used in animal nutrition as a probiotic due to its spore-forming abilities that withstand thermal processing of animal feed [25]. Fungi such as yeasts of the species *Saccharomyces cerevisiae* and *Saccharomyces boullardii* are also used as probiotics [26].

It is crucial for probiotics to survive the gastric passage from oral uptake to the gastrointestinal tract (GI) having a beneficial effect [27]. Although, dead probiotic cells are believed to offer a positive effect on the GI as well, they lose most of their probiotic effect with the loss of viability [28]. Nevertheless, it has been shown that cell compartments such as peptidoglycan or lipoteichoic acids of *L. rhamnosus* GG have an effect on the immune system. Iliev et al. (2005) demonstrated that even the genomic DNA sequence TTTCGTTT of *L. rhamnosus* GG was able to stimulate both murine and human immune cells [29,30].

### Health benefits

Recently, several reviews regarding the potential systemic and GI specific health benefits of probiotics have been published [27,31]. Various probiotic strains are used in pharmaceuticals such as drops or tablets to prevent or treat intestinal diseases by claiming to exploit the antimicrobial activity of some probiotics [32]. Infectious diarrhea, a major problem in both developing and developed countries, is an intestinal disease in which probiotic therapies are utilized [33]. In addition probiotics might also play an important role in human depressive disorders and may influence brain function and behavior [34]. Probiotic effects are restricted to certain strains and are not found broadly within an entire species or genus [35]. Adhesion or aggregation activity is closely related to properties of the bacterial surface layer (S-layer) [36]. The S-layers are self-assembled proteinaceous, planar subunits forming monomolecular-thick crystalline lattices. A few specific functions have been reported for the S-layer such as being a protective coat, a molecular and ion trap and being involved in cell adhesion and surface recognition [36]. These S-layer characteristics are species-specific and are presently not regarded as being genera-specific among the Firmicutes [37].

Probiotic bacteria confer health benefits in diverse ways. Some are able to modify the populations of the gut microbiota by influencing metabolic and nutritional functions of commensal bacteria [38]. Others show indirect and/or direct immune modulating capacities, often by delivering antigens, modulating sensory motor functions, enhancing mucosal barrier functions and/or providing anti-pathogenic effects [38]. Others may prevent metabolic conditions by lowering cholesterol and improving lactose tolerance [39]. Furthermore, probiotics have been reported having positive effects in some gastro intestinal diseases such as inflammatory bowel disease, by being anti-diarrheal and anti-mutagenic [26,40]. Adjuvant effects of probiotics are also used to improve vaccine efficacy [35].

The food industry is promoting these health benefits based on ongoing scientific research. These studies published should help to determine a prophylactic daily dosage to ensure a therapeutic benefit to the consumer [9,31,40]. Many of the strains used like *L. rhamnosus* GG (*Valio* Ltd., Finland), *L. casei* Shirota (Yakult) and *B. animalis* Bb-12 (Chr. Hansen, Denmark) have been studied in detail concerning their beneficial health effects. In case of rotaviral diarrhea, chronic gastrointestinal inflammatory disorders, antibiotic induced diarrhea caused by broad-spectrum antibiotics or diarrhea caused by *Clostridium difficile,* probiotics have been shown to reduce the length and number of episodes [5,41,42]. This is also true for strain *L. casei* DN-114 001 which has been shown to inhibit the interaction of adherent-invasive *E. coli* with intestinal epithelial cells, thereby having a therapeutic effect in Crohn’s disease [43].

In controlled human trials, Ciorba et al. (2012) found that fortified yoghurt containing adequate amounts of viable probiotic bacteria does have beneficial effects on human health [38]. Nevertheless, it is worth mentioning that these effects are also well known for the consumption of fermented food such as red wine, tempeh, red yeast and rice as folk medicine in countries such as India, China and Japan [44].

## Identification methods for members of the genus *Lactobacillus*

### Phenotypic identification

#### Morphology

The identification of strains or species of the genus *Lactobacillus* solely by colony or cell morphology is impossible, however, these characteristics do provide an initial overview of the bacteria present in a product before identifying them using other phenotypic methods or genotyping [45]. Phenotypic methods used either alone or in combination to support cell morphology screening include cell motility testing, Gram staining, and catalase and oxidase reactions [45].

#### API 50 CHL

To differentiate bacterial isolates by their physiological properties various tests are available based on fermentation properties of bacteria. The API 50 CHL system from BioMeriéux (Biomérieux, France) can be used to identify probiotic lactobacilli by testing their fermentation capabilities (fermentative and phenotypic profiling) [46]. The system utilizes a wide spectrum of physiological tests, including substrates covering carbohydrates, heterosides, polyalcohols and uronic acids. Assimilatory, oxidative and fermentative pathways are derived from growth and color changes caused by pH changes.

Globally, many diagnostic laboratories rely on this phenotypic characterization method to identify members of the genus *Lactobacillus* in samples from conditions such as vaginosis. By elucidating the strain’s specific physiological needs this procedure provides an inside view to assimilatory, oxidative and fermentative pathways within one test run. According to the manufacturer’s instructions the results have to be analyzed 48 h after incubation with the APIweb database offered by BioMeriéux (Biomérieux, France) [47].

Reports on the specificity of this method are ambiguous. The most common vaginal bacterial strain, *L. acidophilus*, has been successfully identified by utilizing this method. However, other studies reported limitations of the API 50 CHL system as identical *Lactobacillus acidophilus* strains showed different phenotypic patterns or were nonreactive for all 50 tests included [48]. Boyd et al. (2005) found that one third (33 of 97) of strains identified via API 50 CHL were not specifically identified [49]. A discrepancy of the API 50 CHL results and the known original species was also shown by Nagy et al. (1991) and Alvarez-Olmos et al. (2004) [46,50]. Furthermore, even the APIweb database sometimes caused misidentification or misinterpreted results [49].

A study regarding the isolation of probiotic lactobacilli from fermented traditional food such as kocho (fermented plant powder for bread) and tef flour (whole grain flour) samples in south and south-western Ethiopia compared molecular and phenotypic based methods for the identification of isolated strains and found a discrepancy in the results received from API 50 CHL stripes and Randomly Amplified Polymorphic DNA-PCR (RAPD) cluster analysis [51]. In another study a discrepancy was also detectable when analyzing the phenotypic patterns of members of the *L. casei* group (*L. casei, L. rhamnosus, L. zeae*) with this method [52]. The identification of closely related strains was deemed unacceptable due to the misidentification of a *L. casei* strain as *L. rhamnosus*[52].

In conclusion the API 50 CHL system seems to be appropriate for underlining results based on genomic methods; however, due to the high level of phenotypic variability among lactobacilli this lab-intensive method should not be solely used [49]. In addition, results of API 50 CHL stripes can show acidification processes instead of growth or fermentation processes and even oxygen or a deviation in the density of the bacterial suspension may affect the output. Misidentification and non-interpretable results are clear pitfalls of this method [46,50].

#### Metabolic activity testing using BIOLOG

BIOLOG AN Microplate® (Biolog, Inc., Hayward, CA, USA) was designed to identify members of the genera *Bifidobacterium, Clostridium, Eubacterium, Fusobacterium, Lactobacillus, Lactococcus, Megasphaera, Pectinatus, Pediococcus, Peptostreptococcus, Propionibacterium* and *Weissella*[53]. The BIOLOG system uses tetrazolium and formazan deposition as violet indicators for substrate oxidation in bacterial metabolism processes [54]. These tests are performed simultaneously, and result in a metabolic fingerprint of a strain exposed to 95 different carbon sources [53]. Data are collected by BIOLOG Automatic Reading Instrument and analyzed by BIOLOG MicroLogTM software with the connected database (Biolog, Inc., Hayward, CA, USA) in order to identify the tested strain [53]. The software itself has to be optimized to unambiguously identify a particular species.

However, identification of members of the genus *Lactobacillus* might be difficult. For instance, neither amino acids nor their derivatives are used as a carbon source by *L. rossiae*[55]. De Angelis et al. (2007) mentioned that LAB fermentation capacity varies from very few (e.g. *L. sanfranciscensis*) to a broad range of substrates (e.g. *L. plantarum*) being fermented in BIOLOG’s testing system [56]. In contrast to this, it is possible to analyze physiological abilities within one species. *L. plantarum* strains differed in the fermentation of glycerol, D-malic acid, D-galacturonic acid, inosine, D-sorbitol and D-ketobutyric acid [57].

Although the BIOLOG identification tool offers a wide scope of physiological tests, currently, an unambiguous identification of strains does not seem to be possible [58]. Nevertheless, it appears to be a useful tool to confirm results based on other phenotypic or genotypic tests and to identify the physiological needs and fermentation potential of a particular strain [57,58].

### Physico-chemical identification

#### Fourier Transformation Infra-Red spectroscopy (FTIR)

Since 1911 infrared spectroscopy has been used to analyze biological samples. Between the 1950s and 1960s the popularity of spectroscopy resulted in the development of many new infrared (IR) light-technologies to distinguish microorganisms. Despite this, the approach lost its popularity due to unsatisfactory results [59]. Developed as a result of computer technology and new statistical analysis techniques, Fourier Transformation Infra-Red spectroscopy (FTIR) now presents a much more efficient tool to identify bacteria [60]. In general, IR light is a widely used technique to analyze molecules by identifying their rotation and spinning spectra within seconds [61]. Fourier Transformation Infra-Red spectroscopy (FTIR) uses polychromatic IR light to analyze the rotation and spinning of components in a bacterial sample after continuously firing certain wavelengths of laser light onto it [62].

For instance, in comparison to physiological methods liquid cultures are easy to handle for the analyzation by FTIR. No prior sample preparation is needed and in addition, any physiological state of a sample can be used for rotation and spinning analysis. The spectra are compared to reference data available in the software (Bruker, Billerica, MA, USA) [63].

FTIR technology enables differentiation of bacteria by studying their cell components, fatty acids, membrane and cellular proteins, polysaccharides and nucleic acids [59]. Isolates from diverse food or feed environments such as identification of starter and non-starter cultures from cheese origin can be analyzed [64]. For instance, *L. kefir* shows different surface properties regarding the structure of the S-layer in comparison to other lactobacilli which is important for elucidating their functional abilities (fermentation properties, etc.) [36]. FTIR spectroscopy even allows the identification of intact encapsulated probiotic cells thermally processed in beads being used in environments such as cereals. Starch or sucrose encapsulated probiotic bacteria are able to be analyzed by species-specific proteins, nucleic acids or components of the membrane [64].

The method is rapid, inexpensive, sensitive and allows high throughput analyses for the identification of bacteria [64]. In contrast to other methods such as morphology screening or phenotypic approaches FTIR spectroscopy enables a differentiation of bacteria at the genus, species and strain level (Table 1). However, there are publications available which report about the limitations of using this technique as a single method for identification [65]. Therefore, other methods should be used to confirm FTIR spectrometry results [65]. For instance, in one study comparison of FTIR results with 16S rRNA sequencing confirmed the spectroscopic findings [64].

#### Matrix-Assisted Laser Desorption Ionization - Time Of Flight Mass **S**pectrometry (MALDI-TOF MS)

Each molecule has its own characteristic weight and Matrix-Assisted Laser Desorption Ionization (MALDI) can be used for the characterization of large biomolecules and bacterial proteins with a mass range of 2 kDa and 12 kDa [66]. Astonishingly, whole bacterial cells of overnight cultures can be used for chemotaxonomic classification employing MALDI [67]. This led to a rapid development of MALDI-TOF MS methods for the characterization of targeted or unknown proteins, bacterial RNA and DNA to the level of genus, species, sub-species and strain level (Table 1) [68].

Detecting the protein content of unknown bacteria has to be done by using a matrix of aromatic compounds that are placed and dried on the target before being placed in MALDI-TOF MS aperture [69]. By tearing the matrix sample with a nitrogen laser system (wavelength: 337 nm) molecules are desorbed and ionized in the vacuum [69]. Smaller matrix molecules are heated up by the laser and larger sample molecules are entrained [69,70].

As an example, differentiation of *Lactobacillus casei* and *L*. *paracasei* is challenging as both species belong to the *L. casei* group (*L. casei*, *L. paracasei*, *L. zeae*, *L. rhamnosus*) [71]. As these two species cannot be differentiated by conventional phenotypic methods or by MALDI-TOF MS, a combination of methods (e.g. PCR or 16S Amplified Ribosomal DNA Restriction Analysis [ARDRA]) has to be used for correct identification [72]. Additionally, data of a single *L. casei* strain are available in the BioTyper database (Bruker, USA) cause a misidentification of strains of the same species assigning them to *L. zeae* or *L. paracasei*[66,72]. In contrast, MALDI-TOF MS successfully worked in subspecies determination of two *L. delbrueckii* subsp. *bulgaricus* strains [66].

Within minutes the method enables an extended phenotypic identification of lactobacilli, as the spectrum is embedded in the software of the company [68]. Several commercial software packages are available for microbial species identification such as MALDI Biotyper (Bruker, USA), Axima (Shimadzu, USA), SARAMIS (AnagnosTec, Germany) systems (renamed as VITEK MS [Biomérieux, France]), Andromas (Andromas SAS, France) systems and MicrobeLynx (Waters, USA) [29].

MALDI-TOF MS is a rapid and simple tool for the identification of lactobacilli, although the costs accompanying the purchase and running of a MALDI-TOF MS are extremely high [68,73]. Thus, it is increasingly used in diagnostic laboratories solely or in combination with other methods such as 16S rRNA sequencing to differentiate closely related species [73].

### Genotypic identification

#### Sequencing of 16S/23S-5S rRNA

The 16S rRNA presents the most common target region for phylogenetic analysis at the species level, because sequence data of this region can be used for taxonomic classification. PCR products are easily analyzed using species-specific primers of 16S-23S rRNA and gel electrophoresis [74]. Explicit strain identity is managed by additional sequence analysis. This can be done either by Sanger or pyrosequencing (454), by single-molecule real-time (SMRT) sequencing (Pacific Biosciences, USA), ion semiconductor (Ion Torrent sequencing, Life technologies™, Applied Biosystems, USA), sequencing by synthesis (Illumina, USA) or sequencing by ligation (SOLiD sequencing, Life technologies™, Applied Biosystems, USA) [75]. Likewise, sequence data analysis offers an inside view by BLAST (database of the National Center for Biotechnology Information) or Megalign® alignment suite (Lasergene DNAStar, USA) using the ClustalW algorithm [34]. The intergenic spacer regions (ITS) of 16S-23S-5S rRNA are commonly used to identify LAB, especially lactobacilli [76]. Using colony PCR, a crude cell lysate and species-specific primers targeting the 16S rRNA offers rapid identification of lactobacilli within three hours after isolation (duration for cultivation: 48 h) [76]. A precise assignment of lactobacilli to the level of genus or species is possible utilizing sequences of the 16S-23S-5S rRNA region. Many primers of 16S- or 16S-23S rRNA regions are available discriminating members of the genus *Lactobacillus* at species level using PCR, Denaturing Gradient Gel Electrophoresis (DGGE), RAPD, pulsed-field gel electrophoresis and other methods discussed further on [66,77,78].

However, while 16S/23S-5S rRNA sequencing is useful to identify members of the genus *Lactobacillus* in daily diagnostics, too much time is needed to sequence PCR products and analyze sequence readouts getting reliable results.

#### Quantitative real time Polymerase Chain Reaction (qPCR)

Quantitative real time Polymerase Chain Reaction (qPCR) is a culture-independent and molecular based method. It enables the discrimination of different species and to quantify the amount of bacteria used in a sample. In real time qPCR analysis it is possible using different PCR techniques to measure the amplification process by genus or species-specific primers [79]. For instance, SYBR® Green, TaqMan® labeled primers or molecular beacons are commonly used qPCR techniques.

SYBR® Green is a DNA-binding fluorescence dye which has an affinity to bind to double-stranded DNA (dsDNA) [80]. In contrast to SYBR® Green TaqMan® labeled primers and the molecular beacon technique are probe-based assays marked with a reporter-quencher system [81]. For species-specific detection of a strain a species-specific TaqMan® labeled primer is designed annealing to a sequence internal flanking universal primers.

Molecular beacon probes form hairpins and are not fluorescent in this non-hybridized state [82]. Thus, using one of these methods detection and quantification of a strain is possible without using further post-PCR analyzes steps, if a strain specific sequence is known.

To enumerate bacteria in complex bacterial communities qPCR allows a quantitative approach [83]. Reverse transcription quantitative real time polymerase chain reaction (rT-qPCR) enables the study of growth abilities and activity of bacteria in food estimating their gene expression [84]. Being rapid and culture independent, qPCR is a highly sensitive, specific and accurate method enabling a simultaneous detection and quantification of bacteria in microbial communities [84].

In comparison to culture-based methods qPCR is more rapid and it is possible to detect minor populations of bacteria within dominant populations [85]. Even non-cultivable species (starter cultures of members of the genus *Lactobacillus* in yoghurt containing *Streptococcus thermophilus*) can be detected and quantified using qPCR and PCR [85].

Both, qPCR or rT-qPCR are inexpensive and suitable for daily routine analysis [85]. As post-amplification manipulations are not necessary the risk of contaminations is limited [85]. Under strict and established PCR conditions, C_T_-values and melting curve analysis are tools assuring strain identity. Thus, qPCR are ideal methods for species-specific quantification and identification of bacteria [86].

#### Polymerase Chain Reaction Denaturing Gradient Gel Electrophoresis (PCR-DGGE)

PCR-DGGE is a molecular based method dealing with the analysis of DNA and does not require prior cell cultivation or separation of individual strains. Microbial communities used in probiotic products are easily evaluated with this method. Different DNA sequences have different melting temperatures due to variations in nucleotide composition. Using Polymerase Chain Reaction Denaturing Gradient Gel Electrophoresis (PCR-DGGE) PCR products of the same length can be separated in denaturing gradient gels based on sequence differences. The migration process of the double stranded DNA through the gel stops at its specific melting temperature and separation into single stranded DNA [87]. Then, resulting bands in the gel can be analyzed by comparing them to the control DNA run on the same gel. The intensity of the bands on a DGGE gel is a semiquantitative measure to visualize the dominance of certain strains in the sample over less dominant species [88]. Thus, a limited quantification might also be possible using this approach.

Several publications describe the identification of bacterial communities by PCR-DGGE in cheese [89], sausages [90], wine [91], sourdough [92] and malt whiskey [93]. Many primers are available to amplify sequences of bacteria used in probiotic products, differentiate lactobacilli in GI communities and African and Irish kefir grains.

One drawback to PCR-DGGE is that minor species might not be detectable with this method if they are present in total bacteria populations with less than 1% of the total population [88]. Another drawback is that closely related strains such as *L. casei* / *L. paracasei* might result in equal band sizes in DGGE gel resulting in the misidentification of *L. paracasei* as *L. casei*[94]. In addition, target genes such as *cpn*60 and *rpo*B seem to have a higher discriminative power than 16S rRNA.

However, PCR-DGGE should not be used without additional sequencing of 16S rRNA to assure results [88,95]. In contrast, there might be a lack in designating species by sequencing 16S rRNA PCR products due to high sequence similarity [94]. To avoid false identification a combination of PCR-DGGE and 16S rRNA sequencing of the V3 region might be a potential tool to discriminate species to inter- and subspecies relationships [94]. Additionally, it is time-consuming to identify single bands [96].

#### Randomly Amplified Polymorphic DNA-PCR (RAPD-PCR)

Arbitrary primers are adopted in Randomly Amplified Polymorphic DNA-PCR (RAPD-PCR). These short unspecific primers anneal to multiple random target sequences and lead to band “fingerprints” to distinguish different bacteria [97,98]. A high number of samples can be analyzed within a short time [99].

Several publications report heterogeneities of LAB that can be differentiated by using RAPD-PCR. As an example, this technique was successfully applied to distinguish between *L. helveticus*, *L. sake*, *L. plantarum* and *L. delbrueckii* subsp. *bulgaricus* at both an interspecies and intraspecies level [100,101].

A newly developed Ready-To-Go-RAPD kit decreases the time needed to screen of bacterial communities containing necessary primers [102]. This kit enables the user to follow progress of starter culture activities in vegetable fermentation processes similar to sauerkraut [103].

Even for inexperienced users RAPD is easy to perform and cheap [104]. It does not require prior knowledge of specific sequences to characterize and distinguish bacteria at subspecies levels [102,103]. Gosiewski et al. (2012) observed that the Ready-To-Go-RAPD kit did not discriminate between *L. plantarum* strains of human origin, however, small degrees of variations were detectable *in L. plantarum* strains from plant reference strains [102]. Comparing RAPD and PFGE methods, RAPD has a lower degree of differentiation power among strains such as *L. fermentum* and *L. gasseri* than PFGE [102]. Other authors have reported similar findings indicating that RAPD results are less efficient in comparison to the Pulse-Field-Gel-Elecrophoresis (PFGE) technique [105].

Another pitfall of the RAPD procedure is a reported difficulty in obtaining repeatable results [103]. The procedure can be sensitive to variations in sample preparation resulting in variable results between samples of the same species or strain origin. Therefore, this technique should not be used as a stand-alone method [103]. However, it is a useful technique to confirm results of lactobacilli received by PFGE.

#### Single-strand conformation polymorphism (SSCP)

Frequently, single DNA or RNA strands have a high affinity to form base pairs. However, if no complementary DNA or RNA strand is available, RNA or single stranded DNA form folded conformations with themselves. It is dependent on criteria such as sequence properties and temperature conditions to constitute different conformations. A single mutation in a DNA or RNA strand causes a shift in the single strand modification affecting mobility in gel electrophoresis. If the underlying criteria are known, it is simple to induce self- or folded conformations by single DNA or RNA strands using their DNA fragments in a temperature gradient gel electrophoresis to identify diverse bacterial communities [106].

The Single-strand conformation polymorphism (SSCP) method is also a basis to analyze 16S rRNA [107]. It is a culture-independent tool evaluating LAB communities in food such as raw milk [108], traditional cheeses [109] and fermented fish products [110]. Variations in the V2-V3 region of 16S rRNA are used to identify strains by comparing their SSCP profiles towards reference strains [65]. Obtaining reliable results is possible when combining Restriction Fragment Length Polymorphism (RFLP) for genus identification with SSCP for species identification [65,108]. Additionally, members of the genus *Lactobacillus* are identifiable by combining sequencing and SSCP analysis.

SSCP is a sensitive and accurate procedure to identify bacteria in different environments if methods such as RFLP or sequencing of V2-V3 region of 16S rRNA are used to assure results on species and strain level [20]. Diagnostics using SSCP are less time-consuming and expensive than establishing species-specific primers for PCR. In addition, it is a DNA sequence-based method which does not need any sequence analysis software.

#### Multilocus Sequence Typing (MLST)

Multilocus Sequence Typing (MLST) is based on the analysis of differences in housekeeping gene sequences to reveal relatively distant evolutionary processes to discriminate bacterial strains at the level of intraspecies or subspecies [111]. This method was first described by Maiden et al. (1998). Today, various MLST databases exist such as PubMLST (http://pubmlst.org/) or MLST (http://www.mlst.net/) [112,113].

Several housekeeping genes are used to study intraspecies relationships of LAB (*fus*A, *gdh, gyr*B*, ddl, mut*S*, pur*K1*, pgm, hsp*60*, ile*S*, pyr*G*, rec*A*, rec*G) [102,113,114]. These genes are essential and part of the core genome. Tanigawa et al. (2011) were able to sub-specify species such as *L. delbrueckii* subsp. *bulgaricus* using seven of these housekeeping genes (*fus*A, *gyr*B, *hsp*60, *ile*S, *pyr*G, *rec*A and *rec*G) [113]. However, Adimpong et al. (2013) demonstrated that MLST and splits-decomposition analyses of ribosomal RNA could not detect the correct subspecies level of the used *L. delbrueckii* strain ZN7a-9 T (type strain = DSM 26046 T = LMG 27067 T) [115].

Several publications are available providing information on different gene sequences for identification and classification of members of the genus *Bifidobacterium* (*tuf*, *recA*, *xfp*, *atpD*, *groEL*, *groES*, *dnaK*, *hsp*60, *clpC*, *dnaB*, *dnaG*, *dnaJ1*, *purF*, *rpoC*) [116]. These target genes as well as *pyk* and *tal* have been studied and proven as useful for species and subspecies identification of bifidobacteria [117]. This method was also successfully used to identify *L. casei*[118], *L. plantarum*[119] and *L. sanfranciscensis*[120]. In another publication 16 *L. plantarum* strains were identified by MLST, RFLP and 16S-23S rDNA analysis [119]. In this study MLST offered a much better discriminatory power than RFLP technique utilizing ITS regions showing 14 different allelic combinations within all 16 *L. plantarum* strains [119].

In comparison, the RAPD and the MLST method provide high resolutions [113]. Though, sub-specification of different LAB species in food is possible using MLST technique [113,121]. However, MLST is too laborious and time-consuming to use it for the analysis of a large number of strains in daily diagnostics.

#### Simple Sequence Repeats (SSR)

Loci with high mutation rates located in the genome of strains are useful for bacterial species typing using Simple Sequence Repeats (SSR) [111]. As an example, many SSR tracts are distributed and highly abundant in the genome of *L. johnsonii* NCC533 [111]. These loci are located both in the coding and non-coding regions and deliver the largest number of repeats for genetic characterization [111]. SSR regions of bacteria offer high discrimination power for phylogenetic analysis [111]. In combination with MLST the SSR technique is effective for typing, has high resolution and discriminative power differing between bacterial isolates from same animal species origin to level of subspecies [111].

#### Whole genome sequencing (WGS)

To get an inside view of genetic and structural variations of sequenced individuals for functional and comparative genomic studies whole genome sequencing or high throughput sequencing is the tool of choice [5,122]. PCR amplicons of the DNA of interest are fixed to beads which are sequenced using high throughput sequencing technologies. Next generation sequencing (NGS) enables sequencing processes in parallel producing thousands and millions of short sequence reads simultaneously [123]. Nowadays, high-throughput sequencing technologies are routinely used in biology and medicine to answer important genetically based questions [124]. New approaches were developed by advanced sequencing technologies such as the analysis of metabolic capacities, genome structure and variation analysis of different strains [124]. Being less expensive than formerly used genome sequencing techniques high-throughput sequencing technologies are widely used in industry. Recently, massively parallel sequencing or ultra-high-throughput sequencing (UHTS) offers thousands of sequencing-by-synthesis operations to be run at once [123]. Due to comparably low costs required for UHTS, it is widely used in commercial and academic approaches.

454 sequencing was the first high-throughput sequencing system to become available in the market based on pyrosequencing approach which was developed by Pål Nyrén and Mostafa Ronaghi in 1996 [125]. Expanding the possibilities of Sanger sequencing by using pyrosequencing read outs are simultaneously done by producing light whenever a nucleotide is incorporated [126]. High-throughput sequencing was possible after improving speed and power in computer technologies. The release of light and enhancement of technical analysis even facilitates sequencing in parallel [127]. Additionally, further read outs of the nucleotide structure using electrophoresis are not needed any longer [126].

WGS also offers an overview regarding evolutionary background and divergence of LAB strains belonging to one species [128]. It was shown that LAB genomes have reduced capacity encoding biosynthetic enzymes caused by adaptation to nutrient-rich environments [104]. Approximately, 600–1,200 genes were lost during evolution from their ancestor *Bacillus* including genes for sporulation [128]. Likewise, other genes involved in amino acid transport and peptidases have been duplicated to assure exploring protein-rich environments [128,129].

More and more whole genome sequencing is used to sequence genomes of reference strains using these data for a rapid and secure identification of strains in routine sequence analysis. In addition, sequence data of different mutants of a strain are screened easily assuring an optimal purpose. For instance, WGS revealed that *L. delbrueckii* subsp. *bulgaricus* and *Streptococcus* (*S.*) *thermophilus* should be used in combination as starting cultures to run fermentation processes in milk products [128]. By screening WGS data metabolic capabilities of both species revealed that they are dependent on each other to promote maximum growth potential due to the facility of *L. delbrueckii* subsp. *bulgaricus* to run the complete folate biosynthesis pathway [128]. In contrast, it lacks p-aminobenzoic acid (PABA) production offered by *S. thermophilus*[128,130].

WGS nowadays is commonly used in the food industry as a basis to identify regulatory mechanisms of secondary metabolite overproduction and subsequently improve fermentation processes of products [128]. More rapid fermentation processes reduce incubation time and manufacturers’ costs in creating high quality products [128]. Cogan et al. (2007) demonstrated that genome sequencing offers a fast technique for the analysis of proteolytic abilities of *L. helveticus* CNRZ32 which plays an important role in cheese ripening [131]. Twelve genes were discovered which encode for specific proteolytic enzymes [131].

This method plays a significant role in screening metabolic properties of strains used in food fermentation processes. Thus, it is possible to arrange mutualistic or commensal relationships of starter cultures such as *L. delbrueckii* subsp. *bulgaricus* and *S. thermophilus*[132,133].

In future, WGS will become more important in understanding the evolutionary, functional and physiological aspects of model organisms in medicine, pharmacy and natural sciences due to the fact that costs for WGS will decrease more and more. Thus, strain identification using WGS will increase in future. Even the metabolism and the function of non-cultivatable strains of the human or animal’s microbiome will become decoded. Thus, knowledge about the interaction of microbiota enables abundant possibilities in reducing costs in treatment of e.g. disorders in the gut. To get an inside view of genetic and structural variations of sequenced individuals for functional and comparative genomic studies whole genome sequencing or high throughput sequencing is the tool of choice, although it is a time-consuming method (Table 1) [5,122].

## Discussion & future trends

The aim of this review was to summarize methods and techniques used for the identification of probiotic lactobacilli. It is necessary to detect and to identify probiotics in food due to quality management reasons. Besides advertised strains other probiotic species are found in the same product as they are used as starter cultures running fermentation processes. Therefore, they should be mentioned in the description of the product due to their possible influences on the hosts’ health [31,44,134]. It is necessary to screen products containing probiotics by official authorities or manufacturers thereby assuring the quality of the used probiotic strains (correct and viable strains, correct amount of cells, etc.). As an example, a recommended amount of viable probiotic bacterial cells has been defined between 10^6^ to 10^8^ cfu/ml by different agencies for humans consumption benefitting the immune response for the suppression of allergic and autoimmune disease [9,10]. Methods used for the analysis of probiotic bacteria in food have to be rapid, inexpensive and sensitive with the ability to quantify species of interest.

Some of the previously described methods can be utilized for the identification of lactobacilli at the species level (Table 1). Culture dependent methods (morphology, API 50 CHL, etc.) are well established, however, they are labor-intensive and often do not produce reliable results. Cultivation and isolation of strains from food is generally time-consuming and labor-intensive. The time needed for the designation of lactobacilli species varies between 48 h up to 96 h. It is impossible to identify colonies to the level of genus or species by morphology screening even if colonies are sub-cultivated for additional time periods. A genus, species or sub-species identification is rarely possible. Therefore, culture-dependent isolation only offers a starting point for further investigations of the microbial composition of a product.

It is possible to analyze phenotypic abilities of lactobacilli after isolating them from food. Nevertheless, subspecies detection or quantification is not feasible using API 50 CHL stripes or BIOLOG system. In addition, both methods are time-consuming and labor-intensive and may also lead to misidentified or non-interpreted results in the case of the API 50 CHL system. Another technique for designation of bacteria is analyzing cell wall proteins by Fourier Transformation Infra-Red spectrometry (FTIR). This technology enables a closer insight at the species level instead of morphology screening (level of genus designation, Table 1). When sufficient protein structures will have been included in FTIR databases it is a potential tool to identify bacteria using proteins such as the S-layer of their cell surface. Reliable data of S-layer proteins are known for species used as probiotic additives. These data can be used for strain identification by FTIR which is an inexpensive, rapid and sensitive diagnostic approach. Different FTIR databases are available (http://www.fdmspectra.com/). Limitations of this method have been described when identifying members of the *L. casei* group, a fact which is caused by high genetic similarity of the species belonging to this group.

Another procedure is MALDI-TOF MS being increasingly used for the determination of bacterial species. Both methods – FTIR and MALDI-TOF MS – need approximately the same time for analysis (~ 49 h, Table 1). Furthermore, MALDI-TOF MS permits a rapid and sensitive identification to subspecies level, if protein data are available in a database. However, future research should lead to an increased specificity and sensitivity of both methodologies.

In contrast, culture-independent analysis by utilizing DNA directly isolated from the source of choice is less expensive, less time-consuming than MALDI-TOF MS and enables the user to identify and quantify bacteria down to level of strains (Table 1). A designation of strains within 36 h is possible using specific primers of 16S/23S-5S rRNA and by sequencing PCR products (Table 1). Species identification by sequencing is prolonged for additional 24 h increasing the amount of time needed (Table 1). Nearly the same time is necessary to subtype bacteria from food by MLST.

To our knowledge there is only one method available which offers – besides the identification approach – a second function: quantification of bacterial cells. The qPCR technique delivers all necessary requirements for being useful in daily diagnostic labs. It is rapid, inexpensive, culture-independent and easy to handle. It enables identification and quantification of bacterial cells within one workday (7 h, Table 1) using DNA mixtures directly extracted from food origin (Table 1). In contrast to methods previously described, qPCR does not require a second step of analysis to confirm the results, if primers are validated as specific to their target gene. Thus, qPCR has the potential being one of the most used methods to identify and quantify bacteria within a matrix of interest.

Comparing PCR-DGGE and 16S/23S-5S rRNA PCR plus additional sequencing, PCR-DGGE takes less time (Table 1). While enabling screening of a huge number of samples, it offers limited quantification power. This technique is predestined as being used in daily diagnostics, although, identification of some very closely related species such as *L. casei* / *L. paracasei* is not possible. The RAPD method offers a rapid identification of bacteria. However, Plengvidhya et al. (2004) made clear that it is not useful as a stand-alone method due to lack of reproducibility. Without sequencing, detection of probiotic lactobacilli from PCR amplicons is possible to the species level within 7 h using Single-strand conformation polymorphisms (SSCP). The method itself is accurate and sensitive, even without additional sequencing, although other methods are advised to confirm the results.

A possibility to identify members of the genus *Lactobacillus* may be combining SSR analysis with MLST. However, combining two different methods increases time, labor-intensity and costs. Currently, only few publications are available detecting LAB using SSR technology.

WGS technique itself represents a method which is more and more available on the market due to decreasing costs and their wide range of possibilities by generating whole genome sequencing data. By having these data other techniques become affordable such as designing species-specific primers for qPCR or other molecular based techniques to identify and quantify bacterial strains. In addition, the metabolic potential and abilities of a given strain is available for industrial usage and a much more rapid screening for physiological, evolutionary and functional capabilities is possible. Thus, WGS will become more important for strain identification in future, if costs will decrease steadily. However, it is a time-consuming technique (40–88 h, Table 1), however offers an inside view into the strain’s physiological properties.

In the future, if it is not possible to establish any other identification tool or techniques to analyze probiotics in a much faster way, WGS and real time PCR will become important as rapid tools identifying, screening and analyzing probiotic bacteria compositions. In addition, the availability of both methods supported by decreasing costs will increase their usage within the coming years.

## Competing interests

The authors declare that they have no competing interests.

## Authors’ contributions

SRH designed, structured and prepared the manuscript. In addition, he discussed and interpreted the results. He was also the one who prepared the final manuscript version after discussing it with all the authors mentioned on the manuscript. WV drafted and revised the manuscript critically for important intellectual content and has given final approval of the version to be published. In addition, he took part in writing parts of the manuscript. LHW drafted and revised the manuscript critically for important intellectual content and has given final approval of the version to be published. In addition, he took part in writing parts of the manuscript and in structural arrangement of the paragraphs. SG drafted and revised the manuscript critically for important intellectual content and has given final approval of the version to be published. In addition, he took part in writing parts of the manuscript and in adjusting the text. All authors read and approved the final manuscript.
